# A Case of Hemolytic Uremic Syndrome Due to a Pathogenic Variant in the DGKE Gene

**DOI:** 10.7759/cureus.106224

**Published:** 2026-03-31

**Authors:** Souad Chelghoum, Laurent Mesnard, Nadhir Yousfi, Lionel Rostaing, Ghalia Khellaf

**Affiliations:** 1 Nephrology, UHC Hussein-Dey, Pr Nafissa Hamoud Hospital, Algiers, DZA; 2 Medicine, University of Algiers 1 Benyoucef Benkhedda, Algiers, DZA; 3 Nephrology, Institut National de la Santé et de la Recherche Médicale (INSERM) Sorbonne University, Paris, FRA; 4 Nephrology, Hemodialysis, Apheresis and Kidney Transplantation, Grenoble Alpes University Hospital, Grenoble, FRA

**Keywords:** atypical hemolytic and uremic syndrome (ahus), dgke, eculizumab, genetic diagnostic, targeted treatment

## Abstract

Complement dysregulation is frequently implicated in the thrombotic microangiopathy (TMA) known as atypical hemolytic uremic syndrome (aHUS). Diacylglycerol kinase epsilon (DGKE) mutations encode a non-complement regulatory protein, and pathogenic variants in DGKE define a distinct form of aHUS. Indeed, the DGKEgene encodes a key enzyme involved in intracellular signaling. While eculizumab and other anti-C5 monoclonal antibodies are widely used in complement-related aHUS, their relevance in DGKE-associated forms remains controversial.

We report a case of a patient followed from the age of eight months for suspected aHUS. Genetic testing, performed only at the age of 13 years, revealed a homozygous DGKE mutation (c.412T>C; p.C138R), despite a typical presentation, including hemolytic anemia, thrombocytopenia, and acute kidney injury. Due to severe disease progression and a fatal family history, empirical eculizumab therapy was initiated. Although the treatment resulted in a stable overall clinical status, relapses manifested as isolated nephrotic-range proteinuria (without hemolysis) on two occasions during treatment interruptions. Complement C3 levels remained consistently within the normal range. In this case, eculizumab did not prevent disease relapses, which occurred in the absence of complement activation. The persistence of proteinuria despite C5 blockade further highlights this distinction. The lack of genetic testing options has led to overtreatment in this patient with that type of mutation, with additional costs associated with the drug (eculizumab) and an increased risk of life-threatening infectious complications for the patient.

## Introduction

Acute kidney injury, thrombocytopenia, and non-immune hemolytic anemia are the hallmarks of the rare complement-mediated thrombotic microangiopathy (TMA) known as atypical hemolytic uremic syndrome (aHUS) [1]. Dysregulation of the alternative complement pathway is the primary cause of aHUS, leading to endothelial cell damage, activation, and inflammation through membrane attack complex formation [2,3]. Inherited or acquired abnormalities in the alternative pathway have been identified in approximately 60-70% of patients, including gain-of-function pathogenic variants in genes encoding components of the C3 convertase (C3bBb), such as factor B (CFB) and C3 [4-6]. Anti-complement factor H (anti-CFH) antibodies, linked to homozygous deletions of complement factor H-related 1 and 3 (CFHR1-CFHR3), as well as inactivating variants in genes encoding regulatory proteins of the C3bBb convertase- factor H (CFH), factor I (CFI), and membrane cofactor protein (CD46) - have also been identified. Given recent data, thrombomodulin (THBD) is not considered a gene associated with aHUS [7]. In addition, genetic susceptibility can be conferred by common single-nucleotide polymorphisms (SNPs) or haplotypes in CFH and CD46 [6,8,9]. Beyond complement dysregulation, mostly of dominant transmission, aHUS can also be associated with recessive variants in the diacylglycerol kinase epsilon (DGKE) gene, which encodes DGKE, a protein expressed in endothelial cells and podocytes [10,11]. In the first clinical description, patients with DGKE mutations presented with aHUS in the first year of life; i.e., DGKE mutations were identified in nine of 22 pediatric aHUS patients with disease onset <1 year of age, and 0 of 28 diagnosed after one year of age. Indeed, DGKE mutation was a frequent cause of aHUS in the first year of life, i.e., 27% of all aHUS cases. In addition, the clinical course in DGKE-mutant subjects featured relapsing episodes of HUS before the age of five years. Arachidonic acid-containing diacylglycerols (DAG) activate protein kinase C (PKC), which promotes thrombosis, and DGKE normally inactivates DAG signaling. Thus, the loss of DGKE function results in a prothrombotic state. We report herein a case of a homozygous missense mutation in the DGKE gene associated with aHUS over 12 years.

## Case presentation

At the age of eight months (January 2012), a boy, currently 14 years old, was admitted to the pediatric emergency department with a one-week history of acute gastroenteritis. During the same period, he developed lower limb edema. There was no history of drug exposure. He was born to consanguineous parents with unremarkable medical histories. He has two brothers and three sisters. The family history was notable for a brother and a sister who had died at the ages of three months and 16 months, respectively, from anuria and pulmonary edema.

On admission, the patient appeared pale, hypertensive, and edematous, with ecchymoses on the lower limbs and oligoanuria. Systemic examination revealed no other abnormalities. Laboratory investigations showed serum creatinine of 310 µmol/L, blood urea nitrogen of 45.8 mmol/L, potassium of 5.4 mmol/L, sodium of 137 mmol/L, chloride of 112 mmol/L, bicarbonate of 17 mmol/L, albumin of 2.5 g/dL, hemoglobin of 5.9 g/dL, white blood cell count of 8.4×10⁹/L, platelet count of 97×10⁹/L, haptoglobin <0.08 g/L, and lactate dehydrogenase (LDH) of 985 U/L. Complement analysis revealed complement C3 of 0.97 g/L (normal range: 0.88-2.01 g/L), complement C4 of 0.17 g/L (normal range: 0.15-0.45 g/L), CFH of 210 µg/mL (normal range: 200-400 µg/mL), CFI of 42 µg/mL (normal range: 35-60 µg/mL), CFB of 220 µg/mL (normal range: 200-300 µg/mL), and CD46 cellular expression of 120% (normal range: >90%). Peripheral blood smear revealed schistocytes. Coagulation profile and a disintegrin and metalloproteinase with a thrombospondin type 1 motif, member 13 (ADAMTS13) activity were normal, and the direct Coombs test was negative (Table 1).

**Table 1:** Most relevant laboratory data at first admission. LDH: lactate dehydrogenase

Test name (units)	Observed value	Reference range
Serum creatinine (µmol/L)	310	18-38
Blood urea nitrogen (mmol/L)	45.8	1.8-6.4
Potassium (mmol/L)	5.4	3.5-5.5
Sodium (mmol/L)	137	135-140
Chloride (mmol/L)	112	98-107
Bicarbonate (mmol/L)	17	20-28
Albumin (g/dL)	2.5	3.4-4.2
Hemoglobin (g/dL)	5.9	11.5-13.5
White blood cell count (10^9^/L)	8.4	5-11
Platelet count (10^9^/L)	97	150-400
Haptoglobin (g/L)	<0.08	0.3-2
LDH (U/L)	985	150-353

Urine dipstick analysis revealed hematuria; cytology showed 50 red cells/mm³; proteinuria was 120 mg/mmol. Stool cultures were negative for infectious agents. Renal biopsy could not be performed because of thrombocytopenia, despite adequate blood pressure control.

Given the severity of renal failure, peritoneal dialysis was initiated the same day, along with packed red blood cell transfusions and fresh frozen plasma infusion at a dose of 20 mL/kg. The hypertension was treated with intravenous nicardipine. As eculizumab was unavailable at that time, only supportive therapy was administered. After 45 days of dialysis, renal function improved, as did the hypertension. The peritoneal dialysis catheter was removed, platelet count increased to 170×10⁹/L, hemoglobin rose to 9.5 g/dL, and haptoglobin and LDH normalized. His blood pressure remained stable. The patient was discharged under close follow-up.

One year later, his five-month-old sister was admitted to the pediatric intensive care unit with anuria, hypertension, acute kidney injury, and microangiopathic hemolytic anemia. In addition to supportive measures, she received two doses of eculizumab one week apart. Despite therapies, her condition deteriorated with hemorrhagic purpura and worsening hypertension, leading to death. Following this event, a diagnosis of highly probable aHUS was established in the index patient. Two years later, our patient relapsed with +++ proteinuria and +++ hematuria without impaired renal function. He was placed on nephroprotective treatment (ramipril 2.5-5 mg/day), and eculizumab therapy was initiated. He received a 600 mg intravenous (IV) induction dose followed by 300 mg IV every two weeks for maintenance, according to Food and Drug Administration (FDA) and manufacturer recommendations. Six months after initiation, the patient was stable and in clinical remission. Laboratory parameters were within normal limits except for moderate proteinuria (80 mg/mmol) and hypoalbuminemia (2.5 g/dL). Hemoglobin was 12 g/dL, platelet count 330×10⁹/L, and C3 1.25 g/L (Table 2).

**Table 2:** Laboratory results during follow-up. Renal function, C3 levels, and platelet counts remained within normal ranges during follow-up; conversely, proteinuria was consistently detected, sometimes at high levels.

Test name (units)	Observed value								Reference range
	2014	2017	2020	2021	2022	2023	2024	2025	
Urea (mmol/L)	3.3	4.2	3.8	2.5	3.8	3.9	5.5	6.1	1.8-6.4
Serum creatinine (μmol/L)	46	40	49	55	45	46	43	50	<50
Hemoglobin (g/dL)	12.1	12.2	11.9	12	12.1	12.3	12.2	12.3	>12
Platelets (×10⁹/L)	330	494	298	295	300	298	295	290	150-400
Proteinuria (mg/mmol)	80	50	200	180	34.67	83.17	87.78	50	<20
C3 level (g/L)	1.25	1.216	0.99	1.07	1.09	1.18	0.98	1.02	0.89-1.87

In 2020 and 2021, treatment interruptions of six months at the beginning of each year were followed by relapses manifesting as proteinuria; however, hemoglobin and platelet counts remained within normal ranges (Hb: 12 g/dL; platelets: 295×10⁹/L). Albumin decreased to 2.4 g/dL, and proteinuria increased to 180-200 mg/mmol (Table 2).

Since 2022, continuous therapy with eculizumab has been maintained. There was a reduction of proteinuria and preservation of normal renal function. The patient has remained clinically stable on 600 mg of eculizumab every two weeks, with normal albumin, absence of edema, and stable proteinuria. Serum creatinine has remained within the normal range throughout more than 10 years of follow-up, with no recurrence of hemolytic activity (Table 2).

At the age of 13 years, in 2025, rapid genetic testing based on long-read sequencing using Oxford Nanopore Technologies identified a homozygous missense mutation in DGKE (NM_003647.3), c.412T>C (p.C138R), within a large panel of more than 400 genes related to nephropathies, including aHUS [12]. In particular, no pathogenic variants were detected in the coding regions or intronic regions of ADAMTS13, C3, CD46, CFB, CFH, CFHR1, CFHR2, CFHR3, CFHR5, CFI, MMACHC, PIGA, PLG, THBD, CD59, CR1, CR2, INF2, or MUT.

## Discussion

Genetic testing could not be carried out in 2011 due to a lack of technical facilities and trained genetic staff. To date, our staff has been able to perform a few tests in collaboration with nephrologists and geneticists based in Europe and Canada. The lack of a genetic diagnosis is a major obstacle to the care pathway; however, in recent months, two biology laboratory teams at Ben Aknoun and Rouiba public hospitals in Algiers have acquired a high-throughput sequencer to enable this activity.

Our patient was diagnosed at the age of eight months with aHUS, as well as one of his sisters; this is highly suggestive of DGKE mutation. Once we had made the genetic diagnosis of a homozygous missense mutation in the DGKE gene associated with aHUS, the main therapeutic question was the appropriateness of continuing treatment with the anti-C5 monoclonal antibody eculizumab.

Traditionally, aHUS has been categorized as a complement-mediated TMA. However, the identification of DGKE mutations in 2013 defined a unique subgroup of patients. DGKE is expressed in endothelial cells, podocytes, and platelets. The conversion of diacylglycerol (DAG) to phosphatidic acid plays a crucial role in intracellular signaling. Loss-of-function mutations in DGKE result in sustained activation of the DAG-protein kinase C (PKC) pathway, leading to thrombosis, endothelial activation, and podocyte dysfunction [13]. DAGs also contribute to slit diaphragm integrity in podocytes, and their dysregulation explains the persistent proteinuria observed in DGKE-associated nephropathy [11].

Nearly all patients with DGKE mutations present before the age of five years, with the majority manifesting within the first year of life. Approximately 89% present with aHUS, while 6-20% develop early-onset nephrotic syndrome with renal histology resembling membranoproliferative glomerulonephritis (MPGN) [13,14]. After an initial remission, 64-70% of patients relapse; in the intervals between relapses, microscopic hematuria and chronic proteinuria are frequently observed [11,15]. However, two-thirds of patients with DGKE-HUS had acute kidney injury requiring dialysis, and renal function returned to normal in all but one patient after the first episode [13].

In contrast to aHUS caused by mutations in CFH, CFI, C3, or CFB, DGKE-associated aHUS is not characterized by dysregulation of the alternative complement pathway. This is supported by clinical observations showing that complement levels (C3 and C4) remain within the normal range throughout the course of the disease, including during relapses.

The literature consistently demonstrates that eculizumab is ineffective in this subgroup. Several studies and case series report that patients with DGKE mutations fail to respond to C5 blockade [11,14,16]. In a recent series of 16 patients with DGKE mutations, Brocklebank et al. described the use of eculizumab in six cases [15]. Four patients were successfully weaned off therapy, while one relapsed six weeks after withdrawal and was managed effectively with supportive treatment alone. Improvement observed in some cases following eculizumab administration is generally attributed to concomitant supportive care (plasma therapy, blood pressure control) rather than to C5 inhibition. Historically, plasma therapy, whether plasma exchange or infusion of fresh frozen plasma, has been the most effective strategy for inducing remission. Although its mechanism is not fully understood, it may involve the removal of a prothrombotic factor or the replacement of a deficient plasma factor [13].

In the present case, eculizumab was initiated before genetic confirmation, prompted by strong clinical suspicion of aHUS following the death of the patient’s sister. His subsequent clinical stability under eculizumab may appear misleading. However, several arguments suggest that eculizumab was not responsible as follows: (1) relapses occurred during treatment interruptions in 2020 and 2021, but presented as isolated proteinuria without hemolytic activity (normal hemoglobin and platelet count), (2) complement levels remained normal throughout relapses, indicating a non-complement-mediated mechanism, and (3) proteinuria persisted despite eculizumab therapy, consistent with podocyte injury typical of DGKE nephropathy.

International recommendations, including the 2024 Kidney Disease Improving Global Outcomes (KDIGO) guidelines and the aHUS international working group, clearly state that eculizumab is not indicated for patients with biallelic DGKE mutations [17]. Given the absence of benefit, the high cost, and the increased risk of severe infections, notably a 2,000-fold higher incidence of meningococcal disease, cessation of eculizumab is strongly advised [5]. However, at the beginning, when we had to care for this child because genetic testing was not possible, we placed him on eculizumab therapy.

Discontinuation should be planned under a strict monitoring strategy as follows: rigorous blood pressure control with renin-angiotensin-aldosterone system (RAAS) blockade, monthly to quarterly monitoring of hemoglobin, platelet count, creatinine, proteinuria, and lactate dehydrogenase (LDH). Plasma therapy may be considered as a rescue treatment in the event of clinical relapse, although relapses are uncommon beyond childhood. As illustrated in this case, DGKE-associated aHUS frequently evolves into chronic kidney disease with persistent proteinuria. Importantly, patients with DGKE mutations who progress to end-stage kidney disease (ESKD) have a much lower risk of recurrence after kidney transplantation compared with complement-mediated aHUS. To date, none of the six reported patients with DGKE mutations who underwent transplantation have experienced recurrence [13,18]. Optimal control of hypertension and proteinuria remains the cornerstone of long-term preservation of renal function.

## Conclusions

In conclusion, genetic testing leading to an identification of the DGKE mutation in this patient enabled an accurate etiological diagnosis for aHUS and, most importantly, a fundamental revision of the therapeutic strategy. Both the literature and this case demonstrate that eculizumab is ineffective in DGKE-associated aHUS. Therefore, discontinuation of eculizumab is medically justified and should be accompanied by a management plan focused on strict control of hypertension and proteinuria to preserve long-term renal function.
